# On the Nature and Treatment of Stomach and Urinary Diseases: Being an Inquiry into the Connexion of Diabetes, Calculus, and Other Affections of the Kidney and Bladder, with Indigestion

**Published:** 1841-01-01

**Authors:** 


### On the Nature and Treatment of Stomach and Urinary Diseases: being an Inquiry into the Connexion of Diabetes, Calculus, and other Affections of the Kidney and Bladder, with Indigestion. By *William Prout*, M.D. F.R.S. Fellow of the Royal College of Physicians. Third Edition, much enlarged. London: John Churchill, 1841.

Dr. Prout informs us in a Preface to this third edition of his valuable and well-known work, that it has been re-written, and the materials arranged on principles now for some years before the public. As these principles naturally include almost every disease to which organised beings are liable; with the view of familiarising them, and of rendering the different parts of the volume in some degree independent of each other, the leading points have been purposely repeated—a statement thus made at the outset, to obviate the charge of tautology.

The author, in presenting to the public the *results* of nearly thirty years observation and experience, has still kept in view, as much as possible, the *practical* character of his treatise. All chemical and physiological details, therefore, not urgently required, have been avoided. Such details *may* be given in a future volume. In the mean time, conscious of his fallibility and imperfections, the author invites the candid criticism of the *experienced* chemical pathologist, who alone is capable of appreciating his labours.

Dr. Prout need apprehend no other criticism than a candid one, if indeed he can look for criticism at all. We are inclined to doubt his meeting with any. The modesty and simplicity of the man combine with the scientific character of the work to disarm envy and defeat ill-nature. Few can have the wish, fewer, still, the power to display censoriousness. In reviewing such a book the task is made up of pleasure and profit, for to review, in this case, is to learn.

It is not often that we devote a lengthened space to new editions. But the one now before us is virtually a new work, and one so important in every point of view, so calculated to plant sound doctrines in men's minds, and to widen the sphere of their practical usefulness, that we shall depart from our usual plan, and examine it with some minuteness. We shall at all events endeavour to pick out the fresh matter, and present it to our readers, in this and a subsequent article.

The work consists of an Introduction and two Books.

The Introduction offers us an Outline of the General Physiology and Pathology of Assimilation, and of the Secretion of the Bile and of the Urine.

The First Book, on Functional Diseases, contains five Chapters. The *first*—General Observations on the Pathology of Aqueous Assimilation and Secretion; the *second*—General Observations on the Pathology of Saccharine Assimilation and Secretion; the *third*—General Observations on the Pathology of Albuminous Assimilation and Secretion; the *fourth*—General Observations on the Pathology of Oleaginous Assimilation and Secretion; the *fifth*—General Observations on the Pathology of the Incidental Matters of Organised Bodies.

The Second Book, on Mechanical Diseases, contains seven Chapters. The *first*—Of the Origin and Increase of Calculous Deposites in the Kidneys; the *second*, Of Diseases of the Kidneys, produced by, and liable to be confounded or associated with, Calculus in those Organs; the *third*, Of the Origin and Increase of Calculi in the Bladder; the *fourth*—Of Diseases in the Bladder and its Appendages produced by, and liable to be confounded or complicated with, Vesical Calculi; the *fifth*—Of Hæmorrhage from the Urinary Organs in general; the *sixth*—Of Suppression, Retention, and Incontinence of Urine; the *seventh*—Observations on the Removal of Calculi from the Bladder; comprising Remarks on the Effects of Solvents for the Stone, and on the Operations of Lithotomy and Lithotrity; with a Review of the circumstances which ought to determine the choice of one of these means in reference to the other, or which render all of them dangerous.

It will at once be obvious to those who are acquainted with the former editions of this work how much is new in the present.

The INTRODUCTION of the volume presents us, to borrow Dr. Prout's own phrase, with the *staminal principles* of the work itself. Though it has been present in parts before the public before this, it has never been embodied in so consistent or complete a form. We shall therefore offer what we deem a sufficient account of it.

The first section, which treats of the ultimate composition and structure of organised bodies; and of their general physical characters as dependent on their composition, we pass over, with the simple announcement of Dr. Prout's belief in the existence of an independent vital principle, or principles. All other hypotheses he looks on as absurd.

*Of Alimentary Proximate Principles.*—For many years past, Dr. P. has been accustomed to divide these into four great classes, or groups, denominated the *aqueous*, the *saccharine*, the *albuminous*, and the *oleaginous*.

*Of the Aqueous Alimentary Principle.*—Water, observes Dr. Prout, constitutes not only the medium in which most organic operations are performed; but its elements, either as water or separately, enter into the composition of every living organised being. The subject of water, therefore, in a physiological point of view, may be considered under two heads, as the medium in, or by means of which, all organic operations are performed; and as an alimentary principle.

The proportion of water entering into the composition of organised beings is so remarkable as to appear almost incredible. Not only does the blood contain four-fifths of its weight of water, but even the parts of the body termed *solids*, that is the muscular mass of which animal bodies chiefly consist, contain in reality only about one fourth of solid matter. As an instance in illustration, we may mention a fact stated by Blumenbach, viz. that a perfectly dry mummy of an adult Guanche, in his museum, preserved with all the muscles and viscera entire, did not exceed seven pounds and a half in weight.*

* The original inhabitants of the island of Teneriffe are called Guanches. See the Introductory observations to Blumenbach's Physiology.

The water thus constituting so large a proportion of living animal bodies is the medium by which all vital agencies are performed. In the blood, for instance, the solid organized particles are transported from one place to another; are arranged in the place desired; and are again finally removed and expelled from the body, chiefly by the agency of the water present. Water also imparts to the more solid constituents of the frame that peculiar flexibility and power of extension so characteristic of animal solids.

The quantity of water they possess, is continually changed by the operations of organic bodies. The lungs, the skin, the act of drinking, the kidneys, all affect it. And water, and its elements enter into all organic processes.

*Of Saccharine Alimentary Principles.*—These include a very large class of substances, the general composition of which appears to be similar; that is to say, they consist of a combination of carbon and water in various proportions. The saccharine principles are chiefly derived from the vegetable kingdom, and indeed constitute what may be called, by way of distinction, *vegetable aliments.* As employed by man, some of them exist in the crystallised form, which, from the simplicity of their composition, they readily assume. Of crystallisable saccharine bodies, the chief are *sugar* and *vinegar;* of uncrystallisable or organised bodies, the most remarkable are the different forms of the *amylaceous* or *starchy principle;* the different forms of *lignin* or the *woody principle;* and the different forms of *gum,* or the *mucilaginous principle.*

*Sugar* is the only crystallisable product employed in considerable quantity as an aliment; and by the perfectly healthy stomach seems to be readily assimilated. There are, however, certain states of disease in which this organ appears to lose, in a great measure, the power of assimilating it.

*Vinegar* or *Acetic Acid* is constituted like sugar, from which it is readily produced. It has been employed by mankind, in all ages, in greater or less quantity, as an aliment; that is, substances naturally containing it in small quantity have been employed as aliments; or it has been formed artificially from certain bodies with the view to alimentary purposes. Like sugar, this principle appears to be more difficult of assimilation in its pure or crystallisable form, than in that state of mixture or union in which, for the most part, it naturally occurs.

*Lactic Acid* may be capable of becoming an aliment; but as it is often found unchanged and even developed in the stomach, and indeed in almost all parts of the animal system, it is probably less digestible, and therefore less adapted as an aliment, than the acetic acid.

The remaining saccharine principles are never crystallised, and may be said to be organised.

The *amylaceous* or *starchy* principle, as an aliment, is principally derived from the seeds of the cerealia or corn tribe; but varieties of this principle are found in the roots and other parts of many plants; as arrow-root, from the roots of the maranta tribe: potato-starch, from potatoes; sago, from the pith of the sago palm, &c.

The amylaceous principle is readily assimilated by the healthy stomach, and directly or indirectly forms a constituent of the food of most of the higher animals, as well as of man. It differs, therefore, from sugar, in being a *necessary* article of food, without which animals could not exist; while sugar is not. Hence a much larger quantity of amylaceous matter, than of sugar, can be taken ; and what is a still more decisive fact, the use of this larger quantity of amylaceous matter may be persisted in for an unlimited period, which it appears is not the case with a large proportion of sugar.

Lignin, so far as it has been examined, consists of equal parts of carbon and water. It forms the appropriate food of numerous insects and of some of the lower animals, but of few of the higher classes of animals. The reason of this is probably to be sought for, in their not being furnished with organs proper for comminuting and reducing it ; for when lignin is comminuted and reduced by artificial processes, it is said to form a substance analogous to the amylaceous principle, and to be highly nutritious.

The *gummy* or *mucilaginous* principles form a very numerous class of bodies, nearly allied, if not actually belonging, to the saccharine group, into which they appear to merge by imperceptible grades. As instances of these principles in their well-marked forms may be mentioned, the *sugar of milk*, among crystallised, and *gum arabic* among the uncrystallised bodies.

Dr. Prout doubts whether these are adapted for human aliment, for an unlimited time.

*Albuminous Aliments* are principally derived from the animal kingdom. Hence they are not inappropriately termed *animal aliments*. None of them exist naturally in the crystallised state ; nor can they be made to crystallise by artificial means. Yet certain modifications of them readily assume the crystallised form, and in diseased conditions of the animal economy, in which such modifications occasionally exist in large quantity, they often concrete into masses, and prove a source of secondary disease, as will be shown hereafter.

Dr. Prout describes seriatim the different forms of *albumen—gelatin—fibrin—curd*—and *gluten*. This latter, though chiefly derived from the vegetable kingdom, and, more especially from the seeds of the cerealia, and particularly wheat, is analogous to the albuminous principle, in containing azote, and in being capable of separation into two portions, analogous to gelatine and albumen. Hence the superiority of wheat.

*Oleaginous Aliments.*—However various their forms, these are essentially composed of olefiant gas and water. They are naturally separable into two principal divisions—*fixed and volatile oils*. *Alcohol*, conforming, in composition to the fixed oils, more nearly resembles in properties the volatile. The principal fixed oils employed by man, and derived from the animal kingdom, are *suet*, *lard*, *butter*, *train oil*, &c., while from the vegetable kingdom we have *olive oil*, *nut oil*, and a variety of others.

Such, says our author, are the four great alimentary principles, by which all the higher animals are nourished, and of which their bodies are essentially constituted; and if we regard carbon as the elementary principle by which, *cæteris paribus*, the nutritive powers of three of the alimentary principles are measured or represented, (which, in a certain point of view, may be considered to be the case,) we shall find them to stand in the order in which they have been above described ; that is, the saccharine principles contain on an average from forty to fifty per cent. of carbon ; the albuminous (including azote) from fifty to seventy-five per cent.; and the oleaginous about eighty per cent. of this principle. These staminal principles readily pass under the influence of the organic agents into one another. They are all susceptible of transmutation into new principles according to certain laws: thus the saccharine principle is readily convertible into oxalic acid; or, under other circumstances, into the modification of the oleaginous principle, alcohol. The proportion of these modifications to the staminal principles is extremely limited.

Dr. Prout concludes, that a diet, to be complete, must contain more or less of all the four staminal principles. Such at least must be the diet of the higher classes of animals, and especially of man. It cannot indeed be doubted that many animals, on an emergency, have the power of forming a chyle from one or two of these classes of aliments; but that the higher animals can be so nourished for an unlimited time is exceedingly improbable. Nay, if we judge according to what is known from universal observation, as well as from experiments which have been actually made by physiologists regarding food; we are led to the directly opposite conclusion, namely, that the more perfect animals could not exist on one class of aliments; but that a mixture of three at least, if not of all the four staminal principles, is necessary to form an alimentary compound well adapted to their use.

Dr. Prout cites as an instance—*milk*, with its water, saccharine principle, caseous or albuminous principle, and oily principle—an instance of such combination in an obviously natural description of aliment. It is impossible, however, to name a substance constituting the food of the more perfect animals which is not essentially a compound of three, if not four of the great principles of aliment. This circumstance saves many animals the labour of forming the great proximate principles from their elements. The inferior animals do this; and hence there is a series, from the lowest being that derives its nourishment from carbon and carbonic acid, up to the most perfect animal existing: each individual in the series preferring to assimilate other individuals immediately below itself; but having on extraordinary occasions the power of assimilating all, not only below but above itself, in the system of organised creation.

### Of the Processes of Assimilation.

Dr. Prout divides the processes of assimilation into two great classes, the primary and secondary. The primary assimilating processes comprise the process of digestion, and all the intermediate processes up to sanguification, inclusive; while the secondary assimilating processes comprise the processes by which the different textures of which the living body consists, are first formed from the blood; and afterwards re-dissolved and removed from the system.

In both the processes of assimilation, *water* plays an important part. It enters into the composition of most organised bodies in two separate forms; that is, water may constitute an *essential* element of a substance, as of sugar, starch, albumen, &c., in their *driest* states; in which case the water cannot be separated, without destroying the *hydrated* compound. Or water may constitute an *accidental* ingredient of a substance, as of sugar, starch, albumen, &c., in their *moist* states, in which case, more or less of the water may frequently be removed without destroying the essential properties of the compound. A large proportion of organized bodies contain water in both these forms.

"These," says our author, "containing small proportions of combined water are usually of a firm and stable character, and in common language are said to be *strong* or *high;* while those containing larger proportions of combined water are usually of a delicate and unstable character, and are said to be *weak* or *low;* thus we read of strong and weak sugars, glues, &c., varieties of these principles which are found to owe their peculiar properties to the less or greater proportions of combined water they contain. The processes of converting strong bodies into weak and *vice versâ,* are with difficulty accomplished artificially; for instance, though we can in some respects make a strong sugar weak, in no instance do we appear to be able to reverse the process, and to convert a weak into a strong sugar. As these processes, however, constitute some of the most frequent and important of all the processes of a purely chemical character taking place in organised compounds, it becomes necessary to distinguish them by appropriate appellations; and for this purpose I have been long accustomed to indicate the change of a strong into a weak principle, by the term *reduction,* and *vice versâ,* the change of a weak into a strong principle, by the term *completion*—a nomenclature which will be subsequently adopted throughout these pages, and which the reader is desired to bear in mind." xx.

Dr. Prout views the primary assimilating process as of two kinds. From the stomach to the duodenum inclusive, the operations are all of a *reducing* kind. The *low* and reduced aliments enter the lacteals as chyle. They are now gradually *raised,* and, by the respiratory process, they are rendered *complete.*

The reducing portion of the assimilating process presents three forms.

"First, the stomach has the power of dissolving alimentary substances, or of bringing them into a semifluid condition. This operation seems to be altogether chemical, and probably essentially consists in the combination of alimentary substances with water; that is, in *reducing* the alimentary substances from a high to a low condition. Secondly, the digestive aliments, or the chylous portion of the chyme taken up by the lacteals, though the proportions of its different ingredients may vary, is always essentially the *same* in its composition. The digestive organs, therefore, and more especially the stomach, must possess the power, within certain limits, of changing into one another the simple alimentary principles formerly described. This part of the operations of the stomach appears, like the reducing process, to be *chemical;* but not so easy of accomplishment. It may be termed the *converting* operation of the stomach. Thirdly, the crude and dead aliments undergo changes in the stomach, &c., which render them fit to be brought into contact and even union with the *living* animal body; the stomach and assimilating organs, therefore, must possess the power of *organising* and *vitalising* the different alimentary substances. It is impossible to imagine that such organising agency of the stomach can be chemical. This agency is *vital,* and its nature is completely unknown." xxi.

*The solvent power of the stomach* is described by Dr. Prout. It seems essentially to consist in effecting the more intimate combination of the alimentary principles with water. This is mainly due to the action of the gastric juice. Of this important secretion, says Dr. P. *chlorine,* in some state or other of combination, is an ingredient—it would seem a *necessary*

ingredient; for the secretion in its healthy state always contains more or less of chlorine.

The chlorine of the stomach is exceedingly liable to derangement. Often a large quantity of free muriatic acid is elicited instead, a source both of uneasiness and interference with the reducing process.

The source, says our author, of this chlorine or muriatic acid must be the *common salt* which exists in the blood; to suppose that it is generated, is quite unnecessary. The chlorine, therefore, is separated from the blood, at least in part; and it may be demanded what is the nature of the agency capable of separating the chlorine from a fluid so heterogeneous as the blood? That agency he considers a *modification* of electricity, common electricity being manifestly unequal to the operations effected.

What then becomes of the soda? This, says Dr. Prout, remains behind, or is absorbed into the mass of blood, and a portion of it no doubt is requisite to preserve the weak alkaline condition essential to the fluidity of the blood. But the larger part of this soda is probably directed to the liver, and is elicited with the bile in the duodenum; where it is thus again brought into union with the acid, which had been separated from the blood in the stomach. "Admitting," he adds, "that the decomposition of the salt of the blood, &c. is owing to the immediate agency of a modification of electricity, we have in the principal digestive organs a kind of galvanic apparatus, of which the mucous membrane of the stomach and intestinal canal, generally, may be considered as the acid or positive pole, while the hepatic system may, on the same view, be considered as the alkaline or negative pole."*

Many of our readers are aware that this is an old notion of Dr. Prout's. Fanciful, as no doubt it is, the facts that it attempts to explain subsist, unaffected by its stability or otherwise.

Other acids, and particularly the lactic acid, are occasionally, if not always present during the digestive processes. Dr. Prout's own opinion is that, though frequently present in the human stomach, it is rather to be considered as the result of unnatural irritation, produced by disease, indigestible aliments, &c. than as a healthy product *necessary* to the digestive process. The source of the lactic acid, also, is probably different in different instances. Sometimes it may be derived from the food; at other times, and that most frequently, it seems to be immediately derived, like the muriatic acid, from the blood itself. The lactates exist in the blood, and it may in part be derived from the lactates already formed in that fluid. There is, however, every reason to believe, that when very abundant, it is derived from the decomposition of the blood in the extreme vessels of the stomach, &c.

---

* "This notion or opinion, which was first advanced by me many years ago, seems to have lately received some confirmation from the experiments of Matteuci, who found that when the liver and stomach of a rabbit were connected with the platinum ends of the wires of a delicate galvanometer, a deviation of the needle amounting to fifteen or twenty degrees took place. This action became very feeble or entirely ceased, after the death of the animal: hence he inferred that it depended on the vital action of the organs, and not on the differences of the chemical properties of their secretions. Matteuci, l'Institute, No. 75."

Another acid, an occasional result of unhealthy assimilation, is the oxalic. Its relation to the saccharine elements may sufficiently explain its development. It may also be taken into the stomach as an alimentary matter, for instance, in the stalks of rhubarb, sorrel, &c. Small quantities of oxalic acid thus developed or introduced into the stomach, do not appear to give much uneasiness in that organ; and when introduced, it may in some instances, like other saccharine derivatives, be assimilated. It is secondarily that the oxalic acid proves formidable.

As to the butyric, acetic, carbonic, and other acids occasionally met with during the digestive processes in the stomachs of animals; these are probably in a great degree the results of indigestion, and derived from the mal-assimilation of the alimentary matter.

*Of the Converting Powers of the Stomach, &c.*—Chyle is remarkably uniform, with great varieties of food. Though the albuminous and oleaginous principles need undergo no change, yet the saccharine must. The changes are probably chemical, though Dr. P. admits that we cannot *trace* the conversion of sugar into albumen. Whence comes the azote of the albumen? Dr. Prout believes that it may, in some instances, be derived from the air or *generated;* but that, under ordinary circumstances, it is principally furnished by a highly azotised substance (organised urea?) secreted from the blood, either into the stomach or duodenum, or into both these localities; and that the portion of the blood thus deprived of its azote, is separated from the general mass of blood by the liver, as one of the constituents of the bile; which secretion, as a whole, is remarkably deficient in azote.

Under ordinary circumstances, then, the converting powers of the stomach must essentially consist of the three kinds mentioned, viz. the conversion of saccharine aliments into albuminous and oleaginous principles; the conversion of albuminous principles into oleaginous principles; and the conversion of oleaginous into albuminous principles.

Of these, the first is the most important. As it belongs also to vegetables, it is, perhaps, the lowest step in alimentary renovation. The derangement or partial suspension of the power of converting the saccharine principle in man, not only constitutes a formidable species of dyspepsia; but the unassimilated saccharine matter, in passing through the kidneys, gives occasion to the disease termed *diabetes.*

*Of the Organising and Vitalising Powers of the Stomach, &c.*—Of these we know absolutely nothing, and we need not, therefore, discuss them.

In the *duodenum* the acid of the stomach combines with the alkali of the bile, and the albuminous principles are fully developed, and begin to separate from the excrementitious. In the *lacteals,* the water is gradually removed, and the *completing* process goes on.

### OF THE SECONDARY ASSIMILATING PROCESSES.

Dr. Prout thinks it necessary to enter into an explanation of the sense in which he uses these terms.

"The secondary assimilating processes include two great divisions, which, for the sake of distinction, may be termed the *formative* and the *destructive*. Under the head of the secondary formative assimilating processes, are included the different processes by which the principles of the blood are converted into the different tissues composing animal bodies, as well as the different secretions designed for ulterior purposes in the economy: while under the head of secondary destructive assimilating processes are included the extinction (secondary digestion) of the different tissues of the body, and their conversion, either into new principles designed for ulterior purposes; or into disorganised products designed to be removed from the body, or more frequently into products belonging to both these classes of substances." xxxiv.

Dr. Prout thinks it requisite thus to explain the general principle on which these different processes are conducted.

"When a definite substance, like the albumen of the blood, for instance, is converted into one or more new principles, either the entire elements composing the albumen must be re-arranged so as to produce a principle having new and different sensible properties; or what appears to be infinitely more common, and indeed the rule, the elements of the simple principle must be so arranged as to form *two* (or more) principles, either of which may be said to be *complementary* to the others; that is, the composition of one (or more) of the new principles must be such, as, in conjunction with the remaining principle, will *complete* the albumen, from which all the new principles were originally formed.*

Complementary decomposition is at least of two kinds; a substance may be changed into a new principle designed for ulterior purposes, and another principle designed to be excrementitious; e. g. albumen may be decomposed into gelatine, and hydrated carbon capable of becoming carbonic acid on exposure to air in the lungs. Or a substance may be decomposed into two principles, both of which may be designed for ulterior, or both for excrementitious purposes. Instances, perhaps innumerable, of these two forms of change are constantly taking place in the animal economy; though the first seems more naturally to belong to healthy action; the second to disease. Thus albumen and gelatine are converted into principles, one or both of which are applied to further uses in the economy; whereas, in peculiar states of disease, gelatine in particular appears to be almost wholly converted into some modification of the saccharine principle and urea; both of which from their properties may be considered as excrementitious." xxxv.

As an instance of the decomposition of a principle into three or more complementary principles, Dr. Prout cites gelatine, which is often converted into oxalic acid and the carbonate of ammonia.

Dr. Prout next touches on the *incidental* elements contained in organized bodies. Their incidental mineral matters are supposed to be as fixed and definite, both in their nature and quality, as the essential elements of which such bodies consist. Thus the nervous mass is characterised by the presence of phosphorus; a peculiar tissue intimately connected with the nervous, by

---

* "The part that water plays in complementary decomposition is often very important, and, to prevent misconception, deserves to be noticed. The original substance to be decomposed is often decomposed + or − water; that is, the complementary principles into which a substance is decomposed, do not exactly make up the substance as it usually exists, but the substance, plus or minus, one or more proportions of water. This circumstance is of such frequent occurrence in organic decompositions, as almost to constitute the rule rather than the exception."

the presence of magnesia; certain submucous tissues, by the presence of lime, &c. Dr. Prout agrees with Berzelius in general, that such incidental matters usually exist in their elementary condition in organised products, and not as binary compounds; and that they assume the form of binary compounds or oxides, in which they commonly appear, during the destruction of the organised principle.

Dr. Prout would deduce as important practical inferences from these facts —*that* when incidental mineral matters appear as binary compounds among organised products, the mal-assimilation or destruction of organised tissues is not only indicated; but the exact nature of the tissue thus mal-assimilated or destroyed, may be predicated from the nature of the binary mineral compound.

Dr. Prout now passes to the leading formative and destructive processes of secondary assimilation.

*Gelatification* is that process, by which a certain portion of the fluid albuminous principle of the blood is converted or assimilated into the solid gelatinous tissues of living beings. The gelatinous tissues are the staminal and fundamental ones of the body. The process of gelatification takes place in the extreme capillary blood-vessels, and at the moment when the arterial is converted into venous blood; a phenomenon, therefore, intimately connected with, if not in some degree dependent on, the gelatificating process. Dr. Prout, in his Bridgewater Treatise, attempted to shew that when albumen is converted into gelatine, carbon is eliminated, which carbon (partly perhaps in a hydrated, partly in an oxygenated form,) remains associated with the venous blood till its arrival in the lungs; where, by combining with the oxygen of the atmosphere, it becomes fully oxygenated, and is converted into carbonic acid gas, and in this form makes its escape from the body.

*Albumification*, the process by which the *fluid* albuminous principles of the blood are converted into the *solid* albuminous tissues of living bodies. Albumification, therefore includes albumification properly so called; or that process by which the albumen of the blood is converted into the albuminous textures of the body; and *fibrification*, or that process by which the fluid fibrin of the blood is converted into the solid muscular fibrin of animals. During these processes, *water* must be eliminated.

*The changes undergone by the oleaginous matters* cannot be exactly stated. They undergo certain depurating processes, the separation of water not being the least important.

*Another class of processes* of the formative kind, belonging to the secondary assimilation, some of which are connected more especially with gelatification, others with albumification, are the formation of solid matters, as of bones, horns, hair, &c. &c. While still another class may be supposed to include the different *fluid* secretions derived from albuminous and oleaginous matters, and destined for ulterior purposes; such as the saliva, the different gastric secretions, the spermatic fluid, various oily or resinous secretions, &c.

Dr. Prout passes on to the secondary assimilating process of the *destructive kind.*

*Of the Ulterior Changes of the Gelatinous Tissues.*—Dr. Prout rather thinks that, to a certain extent, these tissues are converted, in the healthy state, into materials of a higher kind. But the more common ulterior alterations are of another description. He has long conceived that one mode in which the gelatinous tissues become effete, is by their conversion into two classes of complementary principles, of which urea, or its equivalent, constitutes one principle; and the saccharine principle in some of its forms, (most frequently in the form of lactic acid,) the other. Both these classes of complementary principles escape by the kidneys in their crystallisable forms, in large quantities; particularly the urea; the lactic acid, escapes, also, from the skin, and from other parts of the body. These changes become mischievous only when excessive, or otherwise abnormal: for example, when the urea is converted into the carbonate of ammonia, or the saccharine principle into oxalic acid, &c.

*Of the Ulterior Changes of the Albuminous Principle.*—They are little understood. He has no doubt that certain portions do become effete, and are eliminated. He thinks that *one* of the crystallisable principles thus formed from albumen during the secondary destructive assimilating processes is lithic acid, most usually in the state of lithate of ammonia. The class of substances complementary to the lithate of ammonia appear to consist of certain ill-defined principles, to be alluded to hereafter—the formation of lithic acid or its compounds in excess, proves a source of disease. And Dr. P. suspects that many formidable diseases are occasionally connected with the destructive mal-assimilation of the albuminous principles; from which principles various matters of a highly deleterious character, and related to the poisonous principle cyanogen, as a basis, may be readily supposed to be eliminated.

*Of the Ulterior Changes of the Oleaginous Principle,* we know even less than of the albuminous. But the large proportion of oleaginous matter which enters into the composition of the nervous mass, shows the important part which oleaginous matters perform in the animal economy; and the disappearance of fat during the process of hybernation, and under many other circumstances, indicates that this principle is most extensively appropriated during the secondary destructive assimilating processes.

*On the General Pathology of the Primary and Secondary Assimilating Processes.*

This section may be said to give their signification and application to the preceding details.

*Primary Mal-assimilation* is mal-assimilation in sanguification. It may occur—*a.* during the digestive processes taking place in the stomach; *b.* during the processes taking place in the duodenum; and *c.* during the subsequent processes taking place in the chyliferous system; or in all these localities simultaneously.

*a.* Mal-assimilation during the *digestive* processes, may more especially belong to the reducing, the converting, or the vitalising functions of the stomach.

Mal-assimilation most frequently commences with derangement of the *reducing* process of the stomach. When any substance incapable of being reduced or dissolved is taken into the stomach, even in its healthy condition, one of the first effects produced is the secretion from the stomach, of a large quantity of acid, of which the *lactic* acid appears to constitute a chief ingredient. The effects of such acidity, immediate and remote, need not be insisted on, and what is not reduced or dissolved, can never be *converted*, much less *vitalised*.

Derangements of the *converting* function occasionally constitute an original disease, the effects of which are still more formidable than those arising from disordered reduction. Thus in diabetic affections, the reducing function of the stomach seems, in some instances, to be almost morbidly active; and farinaceous (and even other) matters are reduced to the condition of low saccharine matter, which the converting function of the stomach is incapable, as in health, of changing into the elements of chyle or blood. The consequence is, that this reduced or dissolved saccharine matter is taken up with the little chyle that may be formed; and after producing various derangements in its transit through the system, is ejected with the urine. Again, the converting process may be wrongly performed; the saccharine matter, for instance, instead of being converted into chyle, may be converted into oxalic, lactic, or other acid and deleterious matters, which may not only produce much local discomfort, but serious disorder in their subsequent passage through the sanguiferous system and kidneys; or even through the bowels.

Or the *vitalising* process may be alone suspended or deranged. Thus, when more food is taken by healthy individuals, than is required for the purposes of the animal economy, such superfluous matters are finally elicited either with the bile; or, in the form of lithate of ammonia in the urine.

*b*, Primary mal-assimilation in the duodenum appears to be more generally the result of mal-assimilation in the stomach, than an original state of disease. The acid matters resulting from the disordered reducing powers of the stomach are not neutralised in the duodenum, and great uneasiness is the result. A portion of the acid is probably absorbed with the chyle.

"In slighter cases of a temporary character, the effects pass off, and all becomes right again; but in severe and protracted cases, arising from derangements of the digestive organs, connected with inveterate constitutional diseases, or from long exposure to strong exciting causes, as malaria, &c. the case is different; and the acid and unnatural matters make their way from the chyliferous system into the abdominal veins; the blood in which vessels often becomes quite black, and sometimes acid. Now as this unnatural blood passes through the hepatic system, the functions of the liver become disordered, and the bile, if not actually rendered acid, at least loses its neutralising properties; and thus the mischief becomes perpetuated. An extreme case is here supposed for the sake of illustration; such, perhaps, as it occurs in the remittent fevers of tropical climates only; but similar phenomena appear in an infinite variety of forms and grades, as the results of mal-assimilation, in all climates." xlv.

It will be observed that Dr. Prout accounts, in this chemical, possibly too simple, fashion, for the furious remittent fevers of the West Indies and of Africa. We cannot, however help suspecting that the operation of malaria is of a deeper and more recondite description. It is right indeed to add that Dr. Prout admits that a variety of other unnatural matters of a complementary character, many of them of an acid, perhaps of a poisonous character, must likewise be generated, and thus contribute, in various ways and degrees, to aggravate the disorder.

*c*. Dr. Prout believes that the consequences of primary mal-assimilation of the lacteal system are most important, especially in the earlier periods of life and adolescence. Dr. Prout advances some opinions on this subject, which we cannot help thinking will take our readers by surprise.

"Now, in early life, under such circumstances, from some causes which I do not profess to explain; but probably from causes connected with original weakness or deficient action of the assimilating organs and of the kidneys; or rather, in short, of the whole system; the imperfectly assimilated chyle in passing through the lacteal system, either does not undergo the necessary changes by which chyle is converted into blood; or is mal-converted into the comparatively insoluble pseudo-albuminous matter of struma; which in passing through the lungs lays the foundation (perhaps at first mechanically) of tuberculous deposition, and future accretion. Whether or not this be admitted, I believe no one will deny who has studied the subject, that about the age we are now considering, the assimilating organs in strumous and consumptive habits are peculiarly deranged; and that great attention to diet, &c., at this age, (when diet is least apt to be attended to, and all sorts of crudities are taken,) will not only sometimes ward off those phthisical attacks, which, when once established, will inevitably run their fatal course; but prevent many nearly allied diseases in after life."*

Setting aside the more severely chemical part of the evidence there do appear some circumstances which favour the notion of the connexion of struma and a tendency to form lithic acid—we allude to the acidity in the primæ viæ, and to the tendency to lithates in the urine of those weakly children who are also subject to struma. But, on the other hand, we are staggered when we contrast the rude health, plethoric habit, and, seemingly, strong constitution of the gouty man, with the delicate organization, weakly frame and valetudinary condition of the scrofulous. At first sight, no two affections can seem more opposed than gout and phthisis. Whether chemistry is in the right, or more simple observation, we shall not venture to pronounce.

*Of Secondary Mal-Assimilation.*—The entire suspension, says Dr. Prout, of the formation of the gelatinous processes from the blood is probably incompatible with life; but the mal-formation and consequent imperfect development of gelatine seems to take place in a variety of degrees and modes. Circumstances also appear to show, that destructive mal-assimilation is equally, if not more frequent than the formative mal-assimilation of the gelatinous tissues; at least as far as regards the class of derangements we are now considering. It is probable, however, that in all instances, both formative and destructive mal-assimilation not only of the gelatinous, but of

---

* "Strumous, lithic acid, and gouty diseases, are all the results of mal-assimilation of the albuminous principle, either primary or secondary; and often gradually run into each other. Thus gout and struma are frequently, if not always, associated; and the gouty chalkstones of old age may be considered as little more than modifications of the scrofulous tubercle of youth, both being alike formed from mal-assimilation of the albuminous principle. Moreover, the offspring of those labouring under gout and struma are (other circumstances being favourable) more subject, during the period of adolescence, to tubercular phthisis, than other individuals. Large deposites of the gouty chalkstone, in middle or advanced age, are often accompanied by incipient disease of the kidneys." xlvii.

all the other tissues, takes place in a greater or less degree simultaneously. As illustrative of these remarks we may observe, that during the secondary formative assimilating processes, instead of gelatine, various unnatural compounds, as sugar, oxalic acid, &c., may be produced; which may not only interfere with the immediate functions of the organs affected, but with the functions of remote organs, as the kidneys, &c., destined to remove such unnatural matters. Again, during the secondary destructive assimilation of the gelatinous tissues, not only the same unnatural matters, as well as others, derived from them, may be generated; but matters of a complementary nature, and of a still more injurious character, may be produced.

Similar remarks, says our author, are applicable to secondary mal-assimilation of the albumificating processes. As instances of the unnatural matters developed during the secondary mal-assimilation of the albuminous tissues; strumous matters, the gouty chalkstone, &c., may be mentioned as developed by formative mal-assimilation; while instead of the lithate of ammonia, which seems to be naturally developed during the destructive mal-assimilation of these tissues, various poisonous principles having relation to the lithic acid in their composition, such as the different compounds of cyanogen, &c., before alluded to, are in some instances undoubtedly generated, and prove the source of formidable secondary derangements, and even of death itself.

Mal-assimilation of the oleaginous principles is of equal, if not greater consequence than the albuminous and gelatinous.

The following remarks of Dr. Prout seem to us extremely just.

"When too much food is taken relatively to the constitution of an individual, either the primary or secondary assimilating processes, or both, may more especially suffer. In some instances, the primary assimilating organs are so weak and so easily deranged, that individuals are constrained to be careful, both with respect to the quantity and quality of their food; and such individuals often escape the more serious and deeper-seated diseases of a secondary kind, arising from excess. On the other hand, there are individuals whose primary organs will permit them to take with impunity enormous quantities of all sorts of matters. In some of these instances, such matters pass off by the bowels very little assimilated; in others, a large portion of them undergo, more or less perfectly, the primary assimilating processes, and are carried into the mass of blood; and individuals in whom this takes place, suffer more especially from derangements of the secondary assimilating processes; as from hepatic congestion, gout, &c., particularly about the middle periods of life, when the consequences of excesses of all kinds begin to be manifested." l.

Dr. Prout points out the connexion between hypertrophy and dropsy. But it does not call for more particular notice. He observes that, when the mal-assimilation is chiefly confined to the gelatinous tissues, the derangements are more especially displayed in the form of certain cutaneous affections, destructive suppuration, or other disease, of the cellular tissues: likewise diabetes, oxalic acid affections, &c. When the development, &c., of the albuminous tissues is chiefly deranged, organic diseases of various kinds connected with these tissues, also lithic acid gravel, &c. are usually the result. Finally, when the mal-assimilation is excessive, and involves the oleaginous in conjunction with the other tissues, the consequence is usually some form of malignant or incurable organic disease.

And, lastly, Dr. Prout insists upon the much more formidable character of secondary diseases depending on secondary mal-assimilation, than of secondary diseases the result of primary mal-assimilation.

We have a section on the general composition and properties of the blood. There is nothing in it to call for notice. He cites the well-known analysis of Lecanu, and remarks upon it:—One of the most obvious facts that first strikes our attention, is the conspicuous place, among the constituent principles of the blood, occupied by two of the great alimentary principles formerly described, viz. the albuminous and the oleaginous principles; while the third, the saccharine principle, is entirely absent. Even the animal (saccharine) principle gelatine, though existing abundantly in various structures, is never found in the blood, nor in any product of glandular secretion. The only constituent of the blood we can suppose to *immediately* represent the saccharine alimentary principle, is perhaps the lactic acid, which Berzelius places among the constituent principles of the blood, and of most animal products soluble in alcohol. M. Lecanu, however, has not specified the lactic acid among the results of his analysis; although there can be little doubt that this acid, if not always present, is at least very rarely absent from the blood.

The next section treats—

*Of the functions of the liver, and of the relation of the bile to the assimilating processes. Of the composition of the bile: and of biliary concretions.*—Dr. Prout points out that the liver is of necessity a great depurating organ—but it also secretes matters absorbed with the chyle—the soda, for example, one. The account of the composition of bile is elaborate and, no doubt, precise. We must refer such of our reders as are anxious for it to the work itself. We may merely observe in the words of Dr. Prout—that the general results of all the analyses of the bile, then, as already stated, are—that the chief organic ingredients, in their general character and composition, partake of the oily character, and contain, probably, at least 80 per cent. of carbon; and—that the saline contents of bile consist principally of soda, which, if not in actual combination with the biliary ingredients, is at least in some other very loose state of combination.

*Biliary Concretions.*—The most frequent are cholesterine in different degrees of purity. Then come gall-stones, consisting of inspissated bile. Biliary concretions composed almost entirely of the colouring matter of the bile are exceedingly rare in the human subject. And, lastly, Mr. Taylor has described an unique concretion, supposed to be biliary, of *stearate of lime.* We proceed to the eighth section—

*Of the functions of the kidneys; and of the relation of the urine to the assimilating processes. Of the composition of the urine; and of urinary calculi.*—Dr. Prout quotes from Rayer, and we feel disposed to quote again, the estimate of the latter gentleman, of the varieties in the weight and size of the organs in the two sexes, and at different ages, &c.

1. That the kidneys of individuals of the same age are never exactly of the same weight.

2. That immediately after birth, the development of the kidneys, though progressive, offers such differences, that in one case the weight of a kidney of an infant of seventeen days old, may be strikingly less than the kidney of another infant of two days old.

3. That in infancy, adult age, and in manhood, the same differences in the weight of the kidney at the same age, are equally observable ; so that the determination of atrophy or hypertrophy of the kidneys is not possible, unless the difference in the weight of the kidneys be very remarkable.

4. That the weight of the kidneys in women, particularly in old age, is less than in men.

5. That the left kidney generally weighs more than the right, at all ages.

6. That in old age the kidneys are generally as heavy as in the prime of life.

7. That when the kidney of an adult or old man, without any remarkable alteration of structure, varies much from three ounces (*French weight,*) it ought to be regarded as having a morbid or an anomalous tendency ; that is, a tendency to atrophy or hypertrophy congenital or accidental.

The *size* of the kidneys is not always proportional to their weight ; but, generally speaking, the kidneys attain their largest size in adult age. As age advances, they usually become less in bulk, but firmer in consistence ; so that, as above mentioned, the weight of the kidneys at this age, does not diminish in proportion to their bulk.

M. Rayer has found the size of the kidney to vary in males, between eighteen and forty-five years of age, from three inches ten lines long, one inch one line wide, and one inch one line in thickness ; to four inches ten lines long, two inches six lines wide, and one inch and nine lines in thickness, *(French measure.)* On the contrary, in males between fifty-five and eighty years of age, the variations have been between three inches six lines long, one inch six lines wide, and one inch thick ; and four inches two lines long, two inches four lines wide, and one inch and five lines thick. In females, the variations have been less remarkable.

Dr. Prout is *now* disposed to think, that if we suppose that the *quantity* of urine varies in this country from 30 ounces in the summer to 40 ounces in the winter, we shall be probably very near the truth, as regards a person in good health, and who does not drink more than the simple wants of nature require.

After long attention to the subject, too, he is of opinion that the standard specific gravity of the urine of a healthy person in the prime of life during the whole year in this country, scarcely reaches 1·020. If, therefore, we estimate the average specific gravity to range from 1·015 in the winter to 1·025 in the summer, we shall be probably very near the truth as regards the generality of well fed individuals, who are ordinarily reputed to be in good health.

Dr. Prout gives a table of the normal and abnormal constituents of the urine, and comments on each in detail. We shall only notice such remarks as may appear peculiar, or important. We introduce one in reference to *urea.*

"Urea was first analysed by myself, about twenty-five years ago ; and from its composition I was satisfied that it might be formed artificially. I made numerous attempts to form it, but did not succeed ; and *the honour of forming the first organic product artificially, is due* to Wöhler. Urea is supposed to be generated during the destructive assimilation of the gelatinous tissues, and probably always exists in the blood in minute quantity. In certain diseases, of the kidney, however, urea exists in the blood in considerable proportions ; a fact established by Dr. Christison, and confirmed by many others." lxxiii.

And we quote too a practical hint that may be serviceable to those who are no great chemists. For *practical* purposes, an *excess* of urea may be shown by putting a little of the urine into a watch-glass, and adding to it carefully about an equal quantity of pure nitric acid, in such a manner that the acid shall subside to the bottom of the glass. The mixture must be kept as cool as possible; and if under these circumstances a crystallised deposite be formed, an excess of urea is indicated. The degree of excess may be inferred, near enough for *practical* purposes, by the length of time which elapses before crystallisation takes place; which may be from a few minutes to two or three hours. The detection of a *deficiency* of urea requires a more elaborate process, which will be found detailed in most recent chemical treatises.

Dr. Prout sticks to his old opinion that the lithic acid exists in the urine, and is held in solution in combination with ammonia, and his reasoning appears conclusive.

Dr. Prout gives a full account of the recent researches of Liebig and Wöhler, on the changes which the lithic acid is capable of undergoing. They are too chemical, however, we might almost add too controversial, for our present purpose. We must content ourselves with stating, as Dr. Prout's conclusions, that the red colour of the urinary sediments is sometimes partially due to the action of the nitric acid on the yellow colouring matters of the urine—in short, that the lateritious and pink sediments of the urine partly depend on the purpurate of ammonia, or some modification of this compound, and partly on the altered yellow principle of the urine; that in different instances, and in different diseases, the two red colouring matters thus produced, are mixed in various proportions; and finally, that the one or the other colouring matter predominates, according to the nature of the disease.

Another point to which we may refer is the *cause* of the deposition of lithic acid in the free state. Now any acid, even the carbonic, added to healthy urine, will throw down the lithic acid, in a crystallised form, a proof that the urine contains no uncombined acid. "When therefore," says Dr. Prout, "the lithic acid is deposited from the urine in the crystallised form, the inference must be, that a free acid exists in the urine; and the question is, what is the nature of this acid? The answer, I believe, to this question is, that in the great majority of instances, the immediate cause of the precipitation of lithic acid gravel is the lactic acid. In some instances, the mineral or other acids may be the *remote* cause of the precipitation; that is, such acids may, from their stronger affinities for the bases present, combine with them and separate the lactic acid, which may thus act immediately as the precipitant, as just stated. In the greater number of instances of lithic acid gravel, however, the lactic acid seems to be actually secreted in excess; either separately, which is comparatively rare; or in a state of combination with urea, which seems to be the rule. Now, as urea has little or no neutralising power, the lactic acid in the lactate of urea exerts its acid powers, and by detaching the lithic acid from its natural state of combination with ammonia, precipitates it in the form of crystallised gravel. As corroborative of this opinion, it may be stated, that the lactate of urea may be sometimes obtained in large quantities from urine depositing lithic acid gravel. This explanation also leads to the explanation of another pathological fact, viz. the frequent presence of sugar in urine depositing lithic acid gravel; and *vice versâ*, the frequent appearance of lithic acid gravel in slight forms of diabetic disease. The lactate of urea, and the saccharate of urea, are in fact but modifications of the same substance, and may both be considered as the representatives of gelatine; the lactic acid being, as we have said, only a modification of the saccharine principle. Hence, by very slight variations in the action of the vital affinities, the acid or the sugar may predominate and give occasion to the phenomena in question. These remarks are also further interesting, since they illustrate certain facts mentioned in other parts of the volume, viz. that the appearance of sugar in lithic acid deposites, is an unfavourable symptom; while, on the other hand, the deposition of lithic acid gravel and of the lithate of ammonia in diabetic urine may be considered as favourable. In the first of these cases, a natural product, the lactate of urea, has given way to an unnatural product, the saccharate of urea; while in the second, the unnatural saccharate of urea, has given way to the natural products, the lactate of urea and the lithate of ammonia,—facts showing that both the gelatinous (saccharine) and albuminous matters are at least partly assimilated."

The *smell* of the urine has never been satisfactorily explained. It is probably connected with some indefinable compound, into which sulphur, phosphorus, and azote, largely enter. The smell of the urine also, as is well known, is liable to be much affected by various articles taken into the stomach, as asparagus, turpentine, &c.

*Sulphur in Cystic Oxyde.*—Dr. Prout admits the justice of the discovery already alluded to in another part of this Journal—that the cystic oxyde calculus contains sulphur. He says—

"I analysed this substance many years ago, and the analysis has been lately confirmed in all respects, except that one half of the matters which I *estimated* to be oxygen, has been proved to be sulphur. I had long suspected that this curious substance contained another principle besides the four usual constituents of organic products; and was about to verify my conjecture, when I heard of the above discovery. I suspected the presence of phosphorus rather than of sulphur." xciii.

*Indigo in the Urine.*—A substance supposed to be Prussian Blue has been met with in the urine. Braconnot has described a substance under the name of *cyanourine*, occasionally found in the urine, and which sometimes tinges it blue. From its properties, this substance appears to be nearly allied to certain vegetable blues; and hence it may, as Mr. Rees has observed, be probably derived from some vegetable substance taken as food. Dr. Prout once met with an instance in which indigo was occasionally voided in the urine, in considerable quantity. The patient was a middle-aged man of a nervous temperament. He was in the habit of taking Seidlitz powders; and the indigo most generally appeared in the urine, in the form of a dark blue sediment, after taking one of these powders. The quantity was so considerable on one occasion, as to allow of its being collected and examined; when it was found to possess all the properties of indigo, and was obtained in a state of purity by sublimation.

*Pus and Mucus.*—Dr. Prout admits the difficulty of accurately distinguishing them. He adds—"Pus, however, when well-marked, may be distinguished from mucus by being essentially composed of *particles*. Hence, when diffused through the urine, which it readily may be, pus, after a time, again subsides to the bottom of the vessel, in the form of a pale greenishyellow pulverulent deposite; and the urine assumes its transparent character; properties by which pus is strikingly contrasted with mucus. Urine containing pus is also almost invariably albuminous; another property by which purulent urine is contrasted with urine merely containing mucus. A third circumstance by which pus and mucus are strongly opposed, consists in the character of the urine. Urine containing pus, particularly when of low or moderate specific gravity, is very often acid, and has little tendency to become alkaline; on the contrary, urine containing much mucus, if not alkaline when passed, speedily becomes alkaline and putrescent. Lastly, pus usually contains a little oily matter, which mucus does not.

The effects of alkalies on pus were, I believe, first pointed out by Mr. Cruickshanks, and these effects are occasionally of considerable importance in a pathological point of view. Thus urine containing both pus and mucus, sometimes becomes alkalescent; and the ammonia evolved converts the pus into a peculiar glairy substance, which imparts to the urine a ropy consistence. This phenomenon, which is not very common, always denotes the presence of a purulent secretion, as well as disease of a mucous membrane; as has been recently noticed by Dr. Babington. I have, however, been acquainted with the fact for many years."

He has seen minute hairs in the urine, when any external source appeared highly improbable, and once he met with them in the pelvis of the kidney after death.

*Substances that do or do not pass through the kidney.*—It may, perhaps, be useful to mention these—useful we mean, in practice. Some substances pass but little changed. Such, for instance, is the hydriodate of potash, which may be detected in a very short time, in the urine of those who have taken it, by the aid of a solution of starch, and a few drops of nitric acid. Other saline matters said to pass through the urine but little changed, are the borate of soda, the alkaline carbonates, the chlorate of potash, the prussiate of potash, the nitrate of potash, the muriate of barytes, &c. &c. On the contrary, the mineral acids, the preparations of bismuth and lead, the oxide of iron, &c. are said by Berzelius and Wöhler not to pass through the kidneys. Among substances of an organic origin, some pass through the system readily, and appear in the urine, while others are decomposed. Of substances passing more or less readily through the system, may be mentioned, the gallic acid, (as in the *uva ursi*, &c.,) also the succinic acid, the carbonic acid? &c. According to some, the citric, malic, and tartaric acids, pass through the kidneys; but this is denied by others, and Dr. Prout thinks with good reason. When combined with alkalies at least, these acids are invariably decomposed in their transit through the system. To the list of substances passing through the system so far as to impart to the urine their peculiar odour, more or less modified, may be added various essential oils and balsams, as turpentine, copaiba, and many others of this class; also the aromatic and colouring principles of coffee, onions, asparagus, &c. With respect to this last class of substances it may be remarked, that the phenomena take place much more readily in dyspeptic, than in healthy individuals. Indeed the odour of almost every thing taken may be detected in the urine of dyspeptic and sedentary persons; and the circumstance may be considered as invariably denoting imperfect assimilation.

Dr. Prout recapitulates in a very useful Table, the details through which he has gone so circumstantially. The table we introduce.

### TABLE,

Exhibiting a contrasted view of the relations between the principles of the blood and the principles of the bile and of the urine, formed either mediately or immediately from the blood.

| **BLOOD** *contains*, In *health*, | In *disease*, | Represented in the *Bile* by — **BILE** *contains*, In *health*. | In *disease*. | Represented in the *Urine* by — **URINE** *contains*, In *health*, | In *disease*, |
|---|---|---|---|---|---|
| Water | | Water | | Watei | |
| Albumen, Fibrin — equivalent to Gelatine, Albumen | Urea, Sugar (Gelatine); Lithic acid? Lithate of soda? (Albumen) | Picromel? | Albumen? | Urea (lactate of) | Carbonate of ammonia, Sugar, Oxalic acid, &c. |
| | | Mucus | | Lithic acid, Lithate of ammonia, Mucus | Purpurate of ammonia, &c. Xanthic oxide, Cystic oxide, Secretion of prostate, Pus, Prussian blue, Indigo? &c. |
| Hæmatosine, Fatty matters | ........................ | Colouring matter? Biliary resin, Cholesterine | | Colouring matter | Colouring matter of bile, Biliary resin, Cholesterine. |
| Lactic acid and its accompanying animal matters, (according to Berzelius.) | .................. | Lactic acid (in combination) and its accompanying animal matters, according to Berzelius. | | Lactic acid and its accompanying animal matters, according to Berzelius. | Free lactic acid. |
| Sulphur, phosphorus, fluorine? in incidental union with animal matters. | | Sulphur, phosphorus, fluorine? in incidental union with animal matters. | | Sulphuric acid, phosphoric acid, fluoric acid, all in combination, as salts. | Sulphur, Phosphorus |
| Muriatic acid in combination as salt. | | Muriatic acid in combination as salt. | | Muriatic acid in combination as salt. | |
| Potash, soda, partly in union with animal matters. | | Potash, soda, partly in union with animal matters and various acids. | | Potash, soda, in combination with acids, as salts. | Free alkalies? Alkaline carbonates. |
| Lime, magnesia, (silex?) in incidental union with animal matters. | | Lime, magnesia, (silex?) in incidental union with animal matters. | | Lime, magnesia, (silex?) in combination with the phosphoric acid. | Lime and magnesia in excess? |

Dr. Prout takes care to observe that the preceding tabular view represents the phenomena as they *generally take place*—the *law* and *not* the *exception.* Of that tabular view he gives a sort of general account which we cannot but look on as important.

"The blood," he says, "contains two different forms of the albuminous principle, one of which, the *albumen,* properly so called, is converted by the secondary assimilating processes into the *gelatinous* (or *saccharine*) and *albuminous tissues;* the other, the *fibrin,* into the *muscular* tissues.* The blood also contains an *oleaginous* principle. The other animal matters present in the blood are ill-defined, and considered by Berzelius to consist chiefly of the débris of the various tissues formed during the secondary assimilating processes. The albuminous principles of the blood, besides the hydrogen, carbon, oxygen, and azote, of which they essentially consist, contain also incidentally, various mineral matters, of which sulphur, phosphorus, iron, calcium, and magnesium are the chief. The oleaginous matters consist of carbon in large proportion, with hydrogen and oxygen, but no azote. The saline matters in the blood consist chiefly of common salt, with soda in some loose state of combination, either with albumen, or other animal matters.

The *bile* contains very little azote; and no principle distinctly known to be analogous to albumen. The peculiar biliary principles (the colouring matter, the biliary resin, and the cholesterine,) contain a large proportion of carbon, and consequently resemble the oleaginous principles of the blood, which, therefore, they probably represent in part. The other animal matters existing in the bile appear to be ill-defined, and to resemble in some degree the ill-defined principles found in the blood. The saline matters in the bile contain relatively a larger proportion of soda than those of the blood; which soda seems to exist in union, partly with the biliary principles, and partly with various acids supposed to be formed from them. The biliary principles, if we except cholesterine in certain forms of disease, do not crystallise, but exist in the bile as first secreted, in imperfectly organised forms.

The *urine* in health contains no albuminous matters; but it contains two principles, urea and lithic acid, in both of which azote is found in large proportion. The urea we suppose to be derived from, or to represent the gelatinous, the lithic acid, the albuminous forms of the albuminous principle. There is no oleaginous principle in the urine; but the colouring matter of the urine (mediately, perhaps, through the colouring principle of the bile) seems to be partly related to the oleaginous principles of the blood on the one hand, and to its colouring matter on the other: neither, if we except the lactic acid, does any form of the saccharine principle exist in healthy urine. The saline matters of the urine differ remarkably from those of the blood and bile. The sulphur and phosphorus which existed in the albuminous principle of the blood are converted in the urine into sulphuric and phosphoric acids. So also, the calcium and magnesium found in the same principles, exist in the urine, as lime and magnesia. The urine also contains ammonia, (derived from the decomposition of urea,) which is entirely wanting in the blood. Hence the number of oxidised and acidified principles found in the urine, as compared with those found in the blood, is remarkable, and places the functions of the kidneys in a very striking point of view." ciii.

Dr. Prout infers that:—

---

* "There is reason to believe, that the colouring principle of hæmatosine, another modification of the albuminous principle, is intimately related to the colouring principles both of the bile and urine."

First. The liver is the organ by which the blood is depurated of the unassimilated and superfluous oleaginous matters; as well as of those portions of the blood deprived of its azote and vitality during the primary assimilating processes.

Secondly. The kidneys are the organs by which the blood is depurated of the unassimilated, superfluous, and effete albuminous principles, as well as the mineral matters incidental to these principles, or which are otherwise derived.

Thirdly. The neutral and alkalescent characters of the bile, and the oxygenated and acidulous characters of the urine, show that the general character of the actions going on in the liver and the kidneys are directly opposed to each other—in short, that the general action of the liver is of a negative, the general action of the kidneys of a positive character; and that one of these two important organs thus antagonistically related to each other, cannot be deranged without deranging the other.

Fourthly. The liver and the kidneys (as well as certain minor glandular apparatus) either in virtue of the polar arrangements above mentioned, or of some other (vital) property, must, in a state of health, possess the *function of selecting* from the blood those peculiar principles adapted for their respective operations; and of producing such further changes in them as the animal economy may require. The changes produced by the liver on the principles to be eliminated by that gland, are in some degree of an organising kind; that is, the principles separated retain some of their vitality for ulterior purposes; while the changes produced by the kidneys on the principles designed to be removed from the system by these glands, are, in a state of health, without exception, of a disorganising kind—that is, everything passed from the kidneys is denuded of its vitality, which is carefully retained as it were in the system. The liver, therefore, may be said to possess an *organising*, the kidneys a *disorganising function*. This deduction is illustrated by what takes place in diseases of the liver and kidneys. Thus, when the liver is diseased, its selecting and organising functions are impaired or lost; and instead of selecting, and further changing into bile those principles, which the welfare of the economy requires should be removed from the blood and employed elsewhere, such principles are retained in the system; or if they do pass through the liver and are separated by that organ, they are imperfectly adapted for their ulterior functions; and thus in both ways great derangements of the health are the consequence. Again, when the kidneys are diseased, their selecting and disorganising functions are impaired or lost; and the deleterious principles in the blood, (e. g. the urea,) are no longer selected in preference for separation from that fluid; while the superfluous or effete albuminous principles, which in the healthy kidney would have been selected and converted into the lithate of ammonia, either remain in the blood, or pass through the kidneys unchanged.

Dr. Prout is confident that these inferences, duly understood and applied, will explain a great many of the phenomena of animal bodies, both in health and in disease.

*Of Urinary Calculi.*—We may just enumerate their varieties. They are, as our readers must be pretty well aware,—the lithic acid calculus; the lithate of ammonia calculus; the oxalate of lime calculus; the cystic oxide calculus; the bone earth, or phosphate of lime calculus; the triple phosphate of magnesia and ammonia calculus; the fusible calculus, or the calculus composed of a mixture of the phosphate of lime and of the triple phosphate of magnesia and ammonia; the alternating calculus (comprising numerous varieties); the mixed calculus; the carbonate of lime calculus; the xanthic oxide calculus; the fibrinous calculus; the prostatal calculus.

The description of these calculi it is unnecessary for us to enter on. The student will be repaid by its examination.

This concludes the "Introduction" to Dr. Prout's volume, and our present notice of it. We have been particular in our account of it; for, although some, indeed most of the views have been before the public at different times already, they have not hitherto assumed so consistent a form, nor have they been developed into so complete a system. And this we will say, that whoever is unacquainted with them is not on a level with the knowledge of the day.

